# Engaging Black or African American and Hispanic or Latino Men Who Have Sex With Men for HIV Testing and Prevention Services Through Technology: Protocol for the iSTAMP Comparative Effectiveness Trial

**DOI:** 10.2196/43414

**Published:** 2023-01-06

**Authors:** Ruth Dana, Stephen Sullivan, Robin J MacGowan, Pollyanna R Chavez, Kristin M Wall, Travis H Sanchez, Rob Stephenson, Lisa Hightow-Weidman, Jeffrey A Johnson, Amanda Smith, Akshay Sharma, Jeb Jones, Marissa Hannah, Monica Trigg, Wei Luo, Joanna Caldwell, Patrick Sean Sullivan

**Affiliations:** 1 Department of Epidemiology Rollins School of Public Health Emory University Atlanta, GA United States; 2 School of Nursing University of Michigan Ann Arbor, MI United States; 3 Division of HIV Prevention Centers for Disease Control and Prevention Atlanta, GA United States; 4 Institute on Digital Health and Innovation College of Nursing Florida State University Tallahassee, FL United States

**Keywords:** HIV self-testing, HIV self-test, prevention, men who have sex with men, internet, mobile phone

## Abstract

**Background:**

Gay, bisexual, and other men who have sex with men (MSM), particularly Black or African American MSM (BMSM) and Hispanic or Latino MSM (HLMSM), continue to be disproportionately affected by the HIV epidemic in the United States. Previous HIV self-testing programs have yielded high testing rates, although these studies predominantly enrolled White, non-Hispanic MSM. Mobile health tools can support HIV prevention, testing, and treatment. This protocol details an implementation study of mailing free HIV self-tests (HIVSTs) nested within a randomized controlled trial designed to assess the benefit of a mobile phone app for increasing the uptake of HIV prevention and other social services.

**Objective:**

This study was a comparative effectiveness trial of innovative recruitment and testing promotion strategies intended to effectively reach cisgender BMSM and HLMSM. We evaluated the use of a mobile app for increasing access to care.

**Methods:**

Study development began with individual and group consultations that elicited feedback from 3 core groups: HIV care practitioners and researchers, HIV service organization leaders from study states, and BMSM and HLMSM living in the study states. Upon completion of the formative qualitative work, participants from 11 states, based on the observed areas of highest rate of new HIV diagnoses among Black and Hispanic MSM, were recruited through social networking websites and smartphone apps. After eligibility was verified, participants consented and were randomized to the intervention arm (access to the Know@Home mobile app) or the control arm (referral to web resources). We provided all participants with HIVSTs. The evaluation of the efficacy of a mobile phone app to support linkage to posttest prevention services that included sexually transmitted infection testing, pre-exposure prophylaxis initiation, antiretroviral treatment, and acquisition of condoms and compatible lubricants has been planned. Data on these outcomes were obtained from several sources, including HIVST-reporting surveys, the 4-month follow-up survey, laboratory analyses of dried blood spot cards returned by the participant, and data obtained from the state health department surveillance systems. Where possible, relevant subgroup analyses were performed.

**Results:**

During the formative development phase, 9 consultations were conducted: 6 in-depth individual discussions and 3 group consultations. From February 2020 through February 2021, we enrolled 2093 MSM in the randomized controlled trial from 11 states, 1149 BMSM and 944 HLMSM.

**Conclusions:**

This study was designed and implemented to evaluate the effectiveness of recruitment strategies to reach BMSM and HMSM and of a mobile app with regard to linkage to HIV prevention or treatment services. Data were also obtained to allow for the analyses of cost and cost-effectiveness related to study enrollment, HIV testing uptake, identification of previously undiagnosed HIV, sexually transmitted infection testing and treatment, and linkage to HIV prevention or treatment services.

**Trial Registration:**

ClinicalTrials.gov (NCT04219878); https://clinicaltrials.gov/ct2/show/NCT04219878

**International Registered Report Identifier (IRRID):**

DERR1-10.2196/43414

## Introduction

HIV testing is the cornerstone of HIV prevention and care and is the starting point for parallel HIV prevention and care continua [1-3]. The Centers for Disease Control and Prevention (CDC) recommends that gay, bisexual, and other men who have sex with men (MSM) test for HIV at least annually and more frequently (eg, at 3- to 6-month intervals) depending on their individual risk factors [4]. However, only about half of the MSM surveyed on the internet and who are not known to be living with HIV meet the minimal recommendation for annual screening [5]. Diagnosing HIV as early as possible, initiating treatment rapidly and effectively to achieve viral suppression, and preventing new HIV infections are the key pillars of the United States Department of Health and Human Services initiative, “Ending the HIV Epidemic in the US” [6]. Thus, interventions are needed for MSM in the United States to increase HIV testing frequency and, for those who are found to be living with HIV, to be promptly linked to HIV care.

Considering the HIV prevention cascade, MSM who obtain a negative HIV test result also have indications for important follow-up prevention services, including sexually transmitted infection (STI) testing [7], provision of prevention commodities (eg, condoms and condom-compatible lubricants), and assessment for pre-exposure prophylaxis (PrEP) initiation [8]. Comprehensive prevention for men who obtain a negative HIV test result should include connecting them with appropriate service providers in the community, identifying sources of commodities if they cannot afford to buy them, and helping them receive other services. The Evaluation of Rapid HIV Self-testing Among MSM Project [9] demonstrated that providing internet-recruited MSM with HIV self-tests (HIVSTs), which could be replenished, resulted in more HIV testing and diagnoses during the study period compared with those who did not receive the HIVSTs. Although there was no statistically significant difference in linkage to care between the 2 arms, men in the HIV self-testing arm reported a lower linkage-to-care rate than those in the control arm. Furthermore, there were no reported differences in the number of sex partners or the number of anal sex partners without condoms among those who received HIVSTs [9]. However, this study had some limitations with external generalizability because Black or African American, non-Hispanic MSM (BMSM) were underenrolled (10%) relative to their representation in the US HIV epidemic (31%), as was the case for Hispanic or Latino MSM (HLMSM; 18% enrolled) relative to the 25% of HLMSM represented in the US HIV epidemic [9-11]. BMSM and HLMSM continue to be disproportionately affected by HIV compared with other groups and, in 2019, accounted for 38% and 35%, respectively, of new HIV diagnoses among MSM in the United States [12]. Research was needed to tailor HIVST delivery to these populations.

To address this gap, we developed a protocol for the Implementation of the Rapid HIV Self-testing Among MSM Project (iSTAMP), with the goal of demonstrating the costs and best practices for reaching BMSM and HLMSM on the internet and providing all participants with free HIVSTs. To ensure prompt linkage to additional HIV testing and care for those with a preliminary positive result and promote linkage to appropriate posttest sexual health care, we provided the intervention arm with a mobile phone app to facilitate linkage to services. We developed methods to enroll BMSM and HLMSM, provide them with HIVSTs, and evaluate the efficacy of a mobile phone app to support linkage to posttest prevention services, including STI testing, PrEP initiation, and acquisition of condoms and compatible lubricants. This paper describes the protocol and enrollment numbers for providing HIVSTs to BMSM and HLMSM enrolled in 11 select US states.

The purpose of this paper is to describe our study procedures and provide recommendations for reaching and distributing HIVSTs to MSM most disproportionately affected by HIV in the United States. In addition, although an HIVST is not designed to link the user to HIV testing and prevention services, we will evaluate the efficacy of a mobile app to increase the linkage to HIV and STI prevention services.

## Methods

### Overview

This study was a comparative effectiveness trial of 2093 cisgender MSM living across 11 states in the United States, including 1149 BMSM and 944 HLMSM. We selected these states based on race- and state-specific estimates of the MSM population size previously developed collaboratively by the CDC and Emory University [13]. We selected the 7 states with the highest populations of BMSM, and the 7 states with the highest populations of HLMSM, because 3 states were included in both lists (Florida, Louisiana, and Mississippi), and 11 implementation states were included in our study (Alabama, California, Florida, Georgia, Louisiana, Mississippi, Nevada, New York, North Carolina, South Carolina, and Texas).

**Figure 1:**
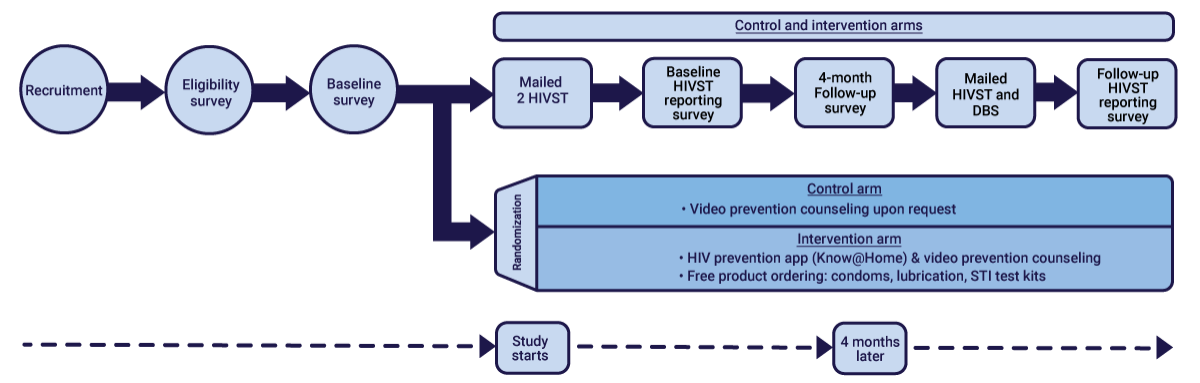
Flow and timeline for Implementation of Rapid HIV Self-testing Among MSM Project, a comparative effectiveness trial of Black or African American and Hispanic or Latino men who have sex with men in the United States (2020-2021). DBS: dried blood spot; HIVST: HIV self-test; STI: sexually transmitted infection.

The study was conducted in 2 phases: phase 1 involved formative discussions with stakeholders to inform the implementation of phase 2. Phase 2 (Figure 1) was a randomized controlled trial, in which participants were recruited through websites and social networking apps. Enrolled participants were mailed 2 HIVSTs (Food and Drug Administration–approved OraQuick In-Home HIV Tests) after providing informed consent, completing a baseline survey, and providing valid contact information. Participants were followed up for 4 months with an additional study assessment at the end of the 4 months. Project staff members discussed the use of HIV surveillance data with the relevant state health departments for the evaluation of the comparative effectiveness of the intervention (access to the Know@Home mobile app) compared with the control (referral to web resources) condition with respect to the linkage of participants to appropriate treatment or preventive services. Phase 2 included obtaining costs for multiple cost-effectiveness analyses related to recruitment; HIV self-testing; and linkage to prevention, treatment, and care services. Recruitment was conducted from February 2020 through February 2021.

### Ethics Approval

This study was approved by the Emory Institutional Review Board (IRB00099710).

### Formative Work

To inform the implementation trial recruitment strategy, study procedures, and linkage-to-care protocol, we conducted individual and group consultations to elicit feedback during the Know@Home study planning and app development phase. This formative work solicited feedback from three core groups: (1) HIV care practitioners and researchers, (2) HIV service organization leaders from study implementation states, and (3) BMSM and HLMSM who live within the implementation study states.

#### One-on-One Consultations With HIV Care Practitioners and Researchers

To gain insight into the nuances of HIV screening among BMSM and HLMSM, 6 HIV care physicians and researchers specializing in working with these populations were invited to participate in one-on-one discussions over video calls. Discussions centered around understanding their experiences of engaging BMSM and HLMSM in HIV research, what advertising venues they found effective for recruiting each population, recommendations for advertising imagery and written content, and approaches to garner trust and foster retention within the project. The notes taken during each discussion were compiled, reviewed by the study team, and assembled into overarching themes. These consultants primarily had experience working with BMSM and had less advice pertaining to HLMSM. There was consensus that dating and hookup apps were the most successful in recruiting BMSM and HLMSM and that images should reflect age, body type, and diversity of skin tone within these populations. Providers working in southern states noted that HIV stigma and distrust of health care and academic institutions were barriers to engaging BMSM in HIV research and prevention and care services. To build confidence, consultants recommended creating relationships with trusted community-based organizations (CBOs) and clinics embedded within these communities. Consultants recommended obtaining support from respected local and state CBOs and clinics to provide study credibility, help keep participants engaged in the duration of the project, and boost linkage-to-care efforts.

#### Group Consultation With HIV Service Organization Leaders

In an effort to help establish relationships with CBOs and ensure practical research deliverables, 5 directors of HIV service organizations were invited to participate in a consultation over a video call. These CBO directors specialized in providing HIV screening and linkage services for BMSM and HLMSM in Alabama, Florida, Mississippi, New York, and North Carolina. The discussions focused on the types of data that would support the creation and implementation of a web-based HIV self-testing program, strategies for reaching BMSM and HMSM, and considerations for scaling such a program to a local organizational level. The notes taken during the consultation were reviewed by the study team, sorted thematically, and assembled into overarching themes. To help create an HIV self-testing program, the CBO directors highlighted their desire for metrics surrounding overall kit distribution, how many results were returned, and how many participants were successfully linked to care. In addition, all directors described the utility of a cost-effectiveness analysis to understand the expenses associated with such a self-testing program in relation to being able to link people who obtained a preliminary positive test result to HIV care. As many of the CBOs had existing laboratory partnerships, the consultants felt that if this program were brought to scale, they would be able to accommodate the processing of returned biospecimens for HIV testing (ie, dried blood spot [DBS] cards) and triple-site bacterial STI testing (ie, pharyngeal swabs, rectal swabs, and urine samples). All directors communicated that their implementation concerns were around staffing and funding, tracking HIVST kits, following up with clients who had a positive test result, and preventing some clients from acquiring more kits than necessary. Finally, CBO directors discussed how having a fully developed protocol for implementing an HIV self-testing program would help them bring a program to scale in their local service provision area.

#### Group Consultations With BMSM and HLMSM

The last element of the consultations sought to receive developmental input from BMSM and HLMSM regarding our recruitment materials, plans, and study procedures. To enlist BMSM and HLMSM, we asked our consultants (ie, HIV physicians, researchers, and CBO directors) in implementation study states to nominate community members who they felt were engaged with HIV and STI issues. Among the nominations offered, 3 HLMSM from California, Florida, and New York and 3 BMSM from California, Florida, and South Carolina participated. Two separate consultations were conducted via video calls—one with HLMSM and another with BMSM. During each consultation, a PowerPoint (Microsoft Corp) presentation was screen shared to elicit feedback on advertising imagery and content, possible recruitment venues, and strategies for disbursing incentives. The discussions also covered participant retention and study implementation design. Notes taken during each consultation were reviewed by the study team and assembled into a set of themes. Across both groups, community members advised using different dating or hookup apps for advertising in each state, based on their varying popularity, in addition to mainstream social media platforms. Retention issues that were cited included participants moving out of eligible study states during the follow-up period and the low monetary incentive for DBS sample collection. Their recommendations for implementation highlighted the importance of discreet shipment packaging, confidentiality of the result-reporting mechanism, linking those with a positive test result to locally trusted CBOs, and privacy of any study app notifications. Finally, the HLMSM group cautioned about the variability of Spanish dialects across the United States when attempting to offer Spanish-translated resources and materials.

During the 2 consultations with BMSM and HLMSM, feedback was obtained on draft advertisement images and taglines to be used for recruitment purposes. Consultants preferred joyous, light-hearted stock images of men to photos that conveyed solemnness. Consultants rated advertisements higher if they showcased everyday situations and “relatable” models that they felt were recognizable within their communities. Images that felt staged, unnatural, generic, or too commercial were ranked lower. Advertisements that portrayed physical affection and intimacy between men were also favored. Men who were shown hugging, kissing, or cuddling portrayed why HIV self-testing may be relevant to the target audience and related to healthy relationships or partner seeking. However, some images where men appeared too serious with their partners were thought to potentially depict intimate partner violence situations. In terms of race and ethnicity, participants gravitated to photos that included Black or African American and Hispanic or Latino men together and represented diverse skin tones. Finally, consultants endorsed tagline text that incorporated puns or turn of phrases such as “It Pays to Know” to more generic messaging such as “Find out your HIV status.”

#### Health Department Engagement

After the study states were selected, we contacted each state health department, beginning with HIV surveillance programs, and had multiple group webinars to share the proposed methods for the study and individual calls with some states to discuss the possibility of matching study participants to HIV surveillance records to ascertain the outcomes of testing.

### Study Procedures

#### Eligibility

Study eligibility and exclusion criteria are listed in Textbox 1. We excluded people who were currently taking PrEP, as they would have been receiving regular HIV testing as part of their clinical care. Participants were required to own an Android or iOS smartphone because components of the trial were delivered via an Android or iOS smartphone app.

**Eligibility criteria for Implementation of Rapid HIV Self-testing Among MSM Project, a comparative effectiveness trial of Black or African American and Hispanic or Latino men who have sex with men in the United States (2020 to 2021).**

- Eligibility criteria:
  Black or African American race, or Hispanic or Latino ethnicity
  Male sex at birth
  Currently identifies as male
  Reported anal sex with at least one man in the past 12 months
  Aged ≥18 years
  Has an Android or iOS smartphone
  Currently resides in a study state (Alabama, California, Florida, Georgia, Louisiana, North Carolina, Nevada, New York, Mississippi, South Carolina, and Texas)
  Willing to download the study mobile app
  Willing to provide valid contact information
  Successful completion of baseline survey

- Exclusionary criteria:
  Currently participating in another HIV prevention research study or program
  Had a bleeding disorder
  Had previously participated in an HIV vaccine study
  Currently taking pre-exposure prophylaxis for HIV prevention
  Reported living with HIV
  Has plans to move out of a study state during the 4-month study period

#### Recruitment, Screening, Consent, and Enrollment

Potential participants were reached through several web-based avenues, hereafter referred to as *recruitment sources*: (1) general, non–lesbian, gay, bisexual, transgender, and queer (LGBTQ)–oriented social networking websites that were not geared toward sex-seeking (eg, Facebook and Instagram); (2) LGBTQ-oriented websites (eg, Queerty or The Advocate); (3) LGBTQ-oriented mobile social networking apps or dating websites geared toward sex-seeking (eg, Jack’d and Adam4Adam); and (4) other (eg, unknown source, referral from participant, and referral from another research study). Advertisements were placed in these web-based venues and directed users to a web page containing basic study information. Interested individuals were required to complete the web-based consent form and screener, and those who were eligible were required to provide contact information. Those who were determined to be ineligible were asked if they could be contacted for future research. We did not indicate why participants were ineligible to prevent unintentional disclosure of the eligibility criteria or repeated attempts to enroll. All participants who completed the screener were directed to web-based resources for HIV prevention and sexual health. After verification and deduplication by study staff, eligible participants were electronically sent links to the main study informed consent form and the baseline survey. The baseline survey was estimated to be completed in approximately 20 minutes.

Completed baseline responses and participant information were reviewed and checked for duplication by the study team. Web-based screener and baseline survey responses were compared to ensure consistency (eg, date of birth, state of residence, and HIV status), and contact information and IP addresses were compared for duplication, as described elsewhere [14]. Participants were sent an SMS text message and were required to respond by verifying their contact mobile number.

After completing verification, participants were enrolled and assigned in a 1:1 ratio to the intervention or control arms using stratified randomization by race or ethnicity, state of residence, and recruitment source (general social media, LGBTQ-oriented websites, LGBTQ mobile apps or web-based dating platforms, and other), totaling 88 strata. Within the strata, participants were randomly assigned to the next treatment allocation from a randomly permuted block sequence (block sizes were 2 and 4).

#### Postenrollment Study Period

Participants in both the intervention and control arms were mailed 2 HIVSTs after completing the baseline survey. Participants were encouraged to use the second HIVST to either (1) test again if the first test did not work (eg, an invalid result or sample collection error) or (2) provide the HIVST to someone they thought may benefit from HIV testing (eg, friend or sex partner). After completing the 4-month follow-up survey, participants were mailed 1 additional HIVST along with the DBS self-collection kit. Participants were encouraged to use the HIVST because the results from laboratory tests conducted on the DBS card could not be returned to participants. When participants used one or more of the study-provided HIVSTs, they were instructed to report each of their HIVST results and upload a photograph of each HIVST device via a secure web-based survey. Participants who gave HIVSTs to someone not enrolled in the study (referred to as *guests*) were advised to have the guest report their result and upload a photograph of their device via a web-based survey that did not collect any identifiable information. Guests were directed to a landing page with a consent form indicating that they were aged ≥18 years before completing an anonymous survey. At any point in the study, the participants and guests were allowed to stop participating.

#### Intervention

This study used the Know@Home app, which was built on the HealthMindr mobile health platform [15-17]. We made administrative changes to the functionality and content to match the study’s design and updated public health messages. The locator tool was updated to national service locators, including HIV.gov and PrEP Locator [18]. Written feedback on content in the app was solicited from community advisory board members, and their input informed changes such as adding more exclamation points to help the messaging seem friendlier, adding information about undetectable=untransmittable, and streamlining the user experience by changing page order and functionality within the app. The app offers a variety of services related to post-HIV test sexual health care, including locators for PrEP services, STI testing, and postexposure prophylaxis services; tools to schedule periodic HIV testing and receive reminders to test; and PrEP and postexposure prophylaxis eligibility screening tools.

#### Incentives

Incentives in the form of e-gift cards were provided to participants for completing various study activities totaling up to US $70 over the 4-month study period. Participants received US $20 to complete the baseline survey, US $30 to complete the 4-month follow-up survey, US $10 to report their HIVST results in the baseline test results survey, and US $10 to complete and return their DBS card to Emory University. Participants could complete a web-based survey for the follow-up HIVST; however, they were not compensated for entering the follow-up HIVST result. Guests who used an HIVST provided by a study participant were not compensated for completing the anonymous web-based survey.

#### Commodity Ordering

Participants in the intervention arm had access to ordering free prevention commodities such as STI self-collection kits, condoms, and condom-compatible lubricants. The delivery of condoms and lubricants was fulfilled by Amazon Fulfillment Services. The delivery of STI self-collection kits was fulfilled by a Clinical Laboratory Improvement Amendments–accredited commercial laboratory. The STI self-collection kits included instructions and materials for participants to collect and return a urine sample, a rectal swab, a pharyngeal swab, a DBS sample, and a microtainer blood sample. The specimens were tested for urethral, rectal, and pharyngeal gonorrhea and chlamydia, and syphilis [19]. Specimens were returned by mail to the originating laboratory, where bacterial STI testing was performed using previously reported methods [20]. Negative STI results were provided to participants through a Health Insurance Portability and Accountability Act (HIPAA)–compliant patient portal, email, or SMS text messages; positive STI results were provided to participants by phone call and the HIPAA-compliant patient portal with referrals to local treatment locations.

#### Results Monitoring

For all study participants, the project staff monitored the HIVST-reporting survey data daily, Monday through Friday, and once on either Saturday or Sunday. The staff members sought to identify HIV test results that were (1) preliminary positive, (2) invalid, or (3) discrepant, meaning the result reported did not match the result image uploaded. When a preliminary positive result was provided, a staff member attempted to contact the participant to assess their emotional state, elicit existing systems of support, locate nearby HIV testing sites, and create a plan for the next steps. When the staff member identified an invalid result, they attempted to contact the participant about either receiving a replacement HIVST in the mail or providing HIV testing site referrals in their local area. After additional testing had been sought, the study staff member followed up with participants to document their corrected test result. If a discrepant result was identified, the study staff member attempted to contact the participant to verify the result entered in the test result survey, describe the source of the discrepancy, and review the test procedures, if needed. State guidelines for reporting an HIV test result to health departments were followed by study staff. As the guest test result survey (designed for individuals who were not enrolled in the study) did not collect identifiable information, the study staff member was unable to contact guests about their reported test results. Study contact information was listed on the guest test result survey, and guests had the option to schedule video counseling with trained study staff.

#### Linkage to Care

The study staff member attempted to contact all participants who reported a preliminary positive HIVST result, up to 10 times, by phone call, encrypted email, and SMS text message. Any unencrypted form of communication (ie, standard email or SMS text message) made no reference to HIV testing or their preliminary positive test result. If not successfully contacted by the third attempt, the study staff member sent a list of free or low-cost local HIV testing sites and care providers through an encrypted email. When the study staff member was able to speak to the participant over the phone, they confirmed that the participant was in a private location where they could talk about their result. If the participant was unable to discuss the results at that time, the study staff member asked for another time to call again. Once privacy had been established, the study staff member started by outlining the purpose of the interaction and then transitioned into inquiring about the participant’s emotional state after receiving the preliminary positive result. The study staff member discussed the importance of having support from someone and addressed HIV disclosure strategies. The study staff member then discussed additional local HIV testing and care options in the context of the participant’s insurance status. Additional referrals offered were case management services, mental health support, HIV support groups, housing programs, food assistance, and transportation services. Agreed-upon referrals were sent via encrypted email that included information about the provider’s website, their hours of operation, and a Google Maps link to the location. After successfully sending additional HIV testing referrals, subsequent contact attempts inquired if an appointment was made or attended, if the preliminary positive result was confirmed, and if the participant was able to initiate treatment. Additional care and support referrals were assessed at each follow-up contact attempt. Participants with a positive additional HIV test result and those who received an HIV diagnosis, had seen an HIV care provider, and received HIV medication were asked if they would like any additional follow-up from the study staff members. Participants were able to opt out of study linkage-to-care support contacts at any time.

Participants in the intervention arm also had the option of ordering STI self-collection kits that included instructions and materials for collecting and returning samples for gonorrhea, chlamydia, and syphilis testing. For any positive bacterial STI test results, the study staff member attempted to contact the participant up to 10 times, following the same procedures as the HIV test results. If after 3 attempts, the participant was not reachable, the participant was sent an encrypted email with a PDF document detailing the laboratory test results and information on several local free or low-cost STI treatment providers. Participants who had specific samples rejected by the laboratory owing to quality or sample collection issues were offered a replacement STI self-collection kit and a list of local STI testing services. Additional contact attempts by the study staff member assessed whether an appointment concerning the STI results had been made or attended and if any additional referrals were needed. Participants who had seen a care provider and received antibiotic treatment had no further contact from the study staff member.

#### Video Counseling

Remotely conducted video counseling services were made available to all iSTAMP participants and guests throughout the study. Staff members who provided video counseling were trained in the state health department HIV testing and counseling and were certified following their state guidelines. In addition, staff members were trained in motivational interviewing techniques for HIV risk behavioral change. Sessions were conducted using HIPAA-compliant Zoom Health with meeting passcodes, waiting room requirements, and end-to-end encryption.

Participants were able to schedule a video counseling session for any reason, but session types were advertised as (1) a pretest session where counselors explained how to collect samples and interpret the HIVST result, and for participants in the intervention arm, the STI collection procedures; (2) a facilitated HIV test session where the participant conducted the HIVST during the video call, discussed current HIV or STI prevention behaviors, interpreted results with the assistance of the video counselor, and then discussed next steps; and (3) a posttest session where participants, who have already completed their HIV self-testing or STI self-collection, discussed their results and received appropriate referrals for treatment or prevention services. Because participants were able to schedule a session at any time during the study without describing their needs beforehand, video counselors were trained to respond to any of these standard scenarios and had a detailed knowledge of study procedures to answer participation-related questions.

After their baseline HIVSTs had been shipped, participants received an email describing the video counseling service and a list of available appointment times for that week. A similar video counseling email was sent 4 months later when another HIVST and DBS card had been shipped to participants. Throughout the project, automated email reminders regarding study activities continued to advertise video counseling services. Participants were able to respond to any study-related communication (ie, phone call, SMS text message, or email) to request a video counseling session. Guaranteed video counselor availability was established during standard business hours, Monday through Friday from 9 AM to 6 PM in the eastern time zone. Participants were also able to schedule during evenings and weekends upon request. The study staff member followed telehealth procedures for client setup, provider setup, troubleshooting, and long-distance referrals described elsewhere [21]. To help demonstrate how to use various biological sample collection materials, the study staff members adhered to existing protocols [22].

### Data Collection

#### Cost-effectiveness Data

A comprehensive cost analysis has been initiated to assess the cost of implementing recruitment strategies and the intervention. A microcosting approach was implemented, along with the standard literature practice of itemizing and assigning a dollar value to each component. Three cost-effectiveness analyses have been initiated from the health care payer perspective (the cost to the party implementing the program), which will include the different cost categories.

First, we obtained the costs incurred for the 3 web-based recruitment venue categories on the outcome of study enrollment (defined as the completion of the baseline survey and downloading of the study app). We collected data on the following cost categories: web-based banner advertisement placement and study staff time to (1) manage enrollment, (2) validate participant eligibility, and (3) monitor and evaluate enrollment data.

Second, we obtained cost data for the 3 venue categories on the outcome of HIVST results returned during the study period and the number of participants with a reactive result. We collected data on the costs of enrollment along with HIVST procurement and shipping, staff or personnel time to manage mailing or return of test kits, study staff time to manage monitoring and evaluation of HIV test result data, and study staff time to provide phone counseling to those who reported a preliminary positive HIV test result.

Finally, we obtained costs data for the intervention on linkage to 3 sexual health services (HIV care visit among those who tested preliminary positive, PrEP discussion with a provider, and STI testing). We collected data on the costs incurred after the participants are provided with HIVSTs and randomly assigned them to 1 of the 2 study arms.

These analyses have included the cost of study staff time for processing orders, contacting participants, and app management or participant registration and are independent of the prerandomization cost-effectiveness analyses; no cost related to recruitment strategies were included.

Cost data were collected by extraction from accounting and budget records, external contracts, and study or personnel time required to manage the outlined tasks. Data were estimated using Clockify (COING Inc), a free software (both web- and mobile based) that allows personnel to track the time spent on daily tasks. To calculate personnel costs, we multiplied these hours by wage rates plus fringe. Only implementation costs (defined as the ongoing costs of delivering the intervention) were considered, and no costs associated with survey or app development, incentives, or research were included.

#### Survey Data

A web-based screening survey collected information to determine eligibility, including demographics, sexual behaviors, state of residence, HIV status, and contact information. Participants, if eligible, then completed a web-based baseline survey that collected information on additional demographic characteristics, sexual behaviors, HIV testing history, future preferences for PrEP use, HIV testing intentions, other health service history, internet and mobile app use, medical mistrust, and health literacy. Participants were prompted by email, SMS text message, or phone to take a web-based follow-up survey after 4 months, which covered the same domains as the baseline survey, with reporting limited to the postbaseline period. Additional topics in the follow-up survey included PrEP use, HIV treatment and care, and time and costs. Participants in the intervention arm answered additional questions in the follow-up survey regarding their experiences using the Know@Home mobile app, and individuals who used the video prevention counseling option were asked about their experiences using this service.

Separate test result–reporting surveys were distributed to participants after the baseline and 4-month follow-up surveys, referred to as the baseline test result–reporting survey and 4-month follow-up test result–reporting survey, respectively. The participant and guest surveys asked about their experience regarding (1) receiving HIVSTs, (2) experience using the rapid test, (3) the result of each HIVST, and (4) distributing HIVSTs.

As participants interacted with the Know@Home app, data about the features used, pages visited, functions used, and time spent were captured by the app and stored in the administrative portal.

#### Laboratory Data

Participants who consented to provide DBS samples for laboratory-based research were provided DBS self-collection kits after completing the 4-month survey. Using the instructions provided, participants prepared and mailed the DBS card for HIV diagnostic testing. Testing included an algorithm with antigen and antibody combination and HIV-1 and HIV-2 differentiation supplemental testing. The DBS samples served both as secondary testing that is more sensitive than the baseline rapid tests and to corroborate or identify new positive HIV results. As a supplemental assessment, participants who returned the DBS card and reported taking HIV medications (PrEP or antiretroviral medications) were sent a microtainer self-collection kit to mail the blood sample to the CDC. The purpose of the microtainer collection activity was to evaluate the feasibility and specimen integrity of self-collected microtainer samples from untrained participants in the United States. The samples were tested for HIV viral load and HIV medications (PrEP or antiretroviral medications).

#### Health Department Surveillance Data

The state departments of public health receive all reports of reactive HIV diagnostic tests, CD4 counts, and viral load tests performed on patients residing in their jurisdictions through systems such as the electronic HIV and AIDS Reporting System. The study staff member requested HIV surveillance data from state departments of public health following the end of a person’s participation in the study. We requested that names and dates of birth of all participants be verified. The goal of this request was to identify participants who received a confirmed reactive HIV test result or entered HIV care for both participants who did or did not report a positive HIV test result during the study.

#### Other Data

Electronic case report forms (CRFs) were used to capture data for validation and enrollment, HIVST result follow-up communications, STI test result follow-up communications, referrals, and video counseling. For those who engaged in video counseling, data on the length and content of sessions were captured and stored in CRFs, excluding identifiable information.

### Planned Analyses

#### Formative Work

In the first part of the study, we characterized the cultural appropriateness, possible web-based recruitment venues, barriers to engaging BMSM and HLMSM in HIV prevention including HIV testing promotion strategies, and additional considerations for the implementation and potential scale-up of mail-out HIVSTs, following conversations with key stakeholders.

In the second part of the study, we initiated several cost-effectiveness analyses and a comparative effectiveness evaluation of the intervention (Know@Home mobile app) on the linkage of participants to appropriate services (HIV treatment, PrEP, STI testing, additional prevention, and social services).

#### Cost and Cost-effectiveness Analyses

In primary analyses, we have initiated an evaluation of the cost and cost-effectiveness of the 2 LGBTQ-oriented recruitment platforms and the general, non–LGBTQ-oriented social networking websites for the following outcomes: (1) enrollment in the study defined as completion of the baseline survey, (2) HIVST results returned within 1 month of enrollment, and (3) identification of established HIV infections (eg, new diagnoses). The secondary analyses consisted of the cost-effectiveness of the 2 randomized study arms for outcomes of linkage to HIV prevention or HIV care services: (1) HIV counseling and treatment for MSM with HIV, (2) PrEP counseling and uptake for MSM who obtained a negative HIV test result, and (3) STI testing and treatment for MSM. The time horizon used was the time from baseline to follow-up, and both costs and effects will be discounted at an annual rate of 3%. Analyses included the overall study population and were performed separately for the 2 MSM populations (BMSM and HLMSM) enrolled.

Standard methods [23,24] for economic analyses have been used for various HIV prevention interventions [24-26]. Cost and outcome data were entered into a standardized Excel spreadsheet with embedded formulas. The net cost of the recruitment strategies and intervention was calculated along with the incremental cost-effectiveness ratio (ICER) for the recruitment strategies compared with the reference (primary aim) or intervention arm compared with the control arm (secondary aim). For example, using ICER to compare the recruitment venues of LGBTQ-oriented mobile apps or web-based dating websites geared toward sex-seeking versus general, non–LGBTQ-oriented web-based websites not geared toward sex-seeking (the reference) on the outcome of enrollment were calculated as follows: [(Cost_sex-seeking apps_ – Cost_reference_)] / [(Enrollment_sex-seeking apps_ – Enrollment_reference_)]. This value yields the incremental cost per participant enrolled. Where no observed differences in the outcomes of interest by recruitment strategy (primary aim) or study arm (secondary aim) exist, then a cost minimization analysis is planned. One-way sensitivity analyses were planned for all input variables to determine the inputs for which the net costs and ICERs were the most sensitive. We explored the effects of realistic implementation scenarios, including health departments with or without the ability to outsource HIV testing, robust eHealth systems, and tech-savvy staff.

#### Usage of Test Kits and Counseling and Linkage to Care

Data on use of HIVSTs were collected in multiple surveys. Information on the use of HIVSTs was provided in test result–reporting surveys during the study, and all participants were also asked about their use of study-provided HIVSTs during the 4-month follow-up survey. The date and result of the most recent HIV test before enrollment in iSTAMP were collected at baseline. Participants were asked to report any additional HIV testing and result that occurred outside the study on their 4-month follow-up survey. For the outcome of HIVST use, we report descriptive statistics about the number of HIVSTs distributed or used during the study period, overall and stratified by race or ethnicity, HIV testing history at enrollment, and recruitment source.

We used data collected on CRFs to calculate the number and proportion of people who used the telehealth counseling provided through the study. We used data from counseling CRFs to describe the main reasons for seeking counseling and the types of services used.

Linkage to care is critical for people diagnosed with HIV. We documented the proportion of participants who tested positive for HIV that were linked to care and described the distribution of time from receiving a positive test result to linkage to care, as previously described [9]. We used 3 sources of data to describe linkage to care. First, we used the following data from the study surveys: participants who reported a positive test result were asked if they have seen an HIV care provider and the approximate date of their appointment with that provider. Second, we used notes from CRFs prepared by study staff who talked with participants and assisted with linking them to HIV care. Not all participants requested such assistance, so not every participant who received a positive result has a linkage or counseling CRF. Finally, we have worked with the 11 state health departments to create plans to match our participants to the state surveillance registries. This would allow us to assess the occurrence and timing of additional HIV testing and viral load evaluation and to determine the dates of these services as a marker of linkage to care. We have functioned as a testing provider and reported HIV diagnoses as required (some states require reporting of positive HIV self-test results). We provided feedback to testing providers about the linkage and care outcomes of people that the providers diagnose as a routine function of surveillance programs. Furthermore, we obtained informed consent from the participants to allow for such linkage.

#### Receipt of Posttest Services by Allocation to App

HIV testing is the entry point for multiple care continua; for those who received a positive test result in this study, their linkage to care was assessed. We assessed the receipt of HIV and STI services during the follow-up period for those who received a negative result or who do not report HIV testing. We also determined whether randomization to the intervention arm (Know@Home app built on the HealthMindr platform [15,16]) was associated with a higher percentage for three outcomes measured at month 4: (1) testing for bacterial STIs in the previous 4 months; (2) receipt of condoms and condom-compatible lubricants in the prior 4 months; and (3) linkage to HIV (PrEP or antiretroviral therapy) services during the study follow-up period. We used an α value of .05 to designate statistically significant intervention effects. Given that we had prespecified 3 primary outcomes, adjustment for multiple comparisons was not necessary [27].

## Results

### Key Themes in Formative Work

Key themes in formative work among HIV providers and researchers (N=6) indicated that dating and hookup mobile apps have previously been successful in recruiting BMSM and HLMSM for research studies. In addition, HIV stigma and distrust of health care and academic institutions may act as barriers to engaging with these populations. Among HIV organization directors (n=5), primary themes included their desire for cost-effectiveness data for possible implementation of HIV self-testing services and concerns about staffing, funding, and linkage to care for clients who use an HIVST. Among the BMSM (n=3) and HLMSM (n=3) community members, the central findings consisted of their advertising preferences for noncommercial images, diverse skin tones, and joyous and everyday scenes, accompanied by text that incorporated puns.

### Progress to Date

**Figure 2:**
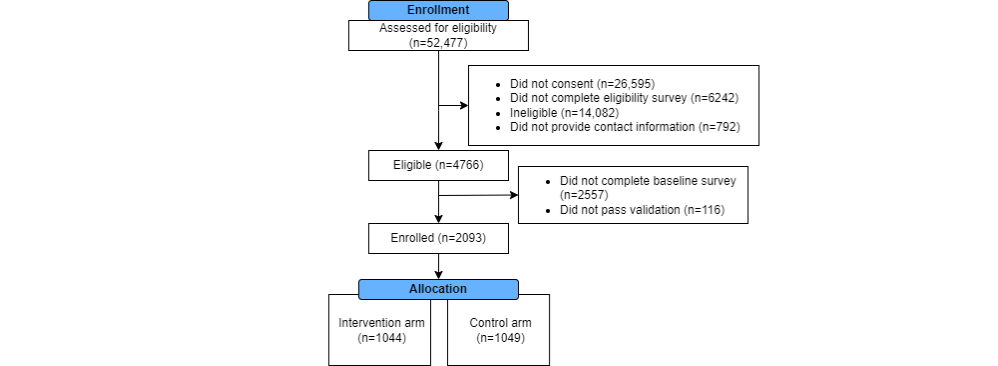
CONSORT (Consolidated Standards of Reporting Trials) diagram for Implementation of Rapid HIV Self-testing Among MSM Project, a comparative effectiveness trial of Black or African American and Hispanic or Latino men who have sex with men in the United States (2020-2021).

iSTAMP began enrollment in February 2020 and concluded in February 2021, with participant follow-up data completed in September 2021. Of the individuals assessed for eligibility with screening questions, 9.08% (4765/52,481) were considered eligible to join (Figure 2). Of the MSM who consented, 43.92% (2093/4765) completed the baseline survey, passed the validation process, and were enrolled, of whom 45.10% (944/2093) were HLMSM and 54.90% (1149/2093) were BMSM. We randomly assigned 49.88% (1044/2093) participants to the intervention group and 50.12% (1049/2093) to the control group.

To examine enrollment proportions relative to state demographic data, we compared participant enrollment numbers with the 2018 Census [10], which estimated the male population in each state, stratified by race or ethnicity (Table 1). Census data were categorized for comparison by including only those who selected single-race Black or African American, non-Hispanic, while iSTAMP allowed for participants included in the Black or African American, non-Hispanic strata to select multiple races.

**Table 1:** Enrolled study population compared with census-estimated population from 2018 by race or ethnicity for Implementation of Rapid HIV Self-testing Among MSM Project, a comparative effectiveness trial of Black or African American and Hispanic or Latino men who have sex with men in the United States (2020 to 2021).

State	Black NH^a^ MSM^b^ enrolled (n=1149), n (%)	Black NH men^c^ in the state population (11 states; n=8,046,742), n (%)	Hispanic MSM enrolled (n=944), n (%)	Hispanic men in the state population (11 states; n=13,895,483), n (%)
Texas	205 (17.84)	1,220,831 (15.17)	253 (26.8)	3,842,932 (27.66)
South Carolina	42 (3.66)	465,763 (5.79)	14 (1.5)	102,440 (0.74)
North Carolina	102 (8.88)	774,275 (9.62)	46 (4.9)	319,908 (2.30)
New York	137 (11.92)	1,008,059 (12.53)	135 (14.3)	1,330,085 (9.57)
Nevada	18 (1.57)	101,306 (1.26)	17 (1.8)	300,733 (2.16)
Mississippi	43 (3.74)	375,276 (4.66)	7 (0.7)	37,118 (0.27)
Louisiana	55 (4.79)	509,679 (6.33)	12 (1.3)	89,693 (0.65)
Georgia	242 (21.06)	1,121,964 (13.94)	57 (6.0)	344,266 (2.48)
Florida	136 (11.84)	1,157,557 (14.39)	112 (11.9)	2,049,528 (14.75)
California	109 (9.49)	870,988 (10.82)	277 (29.3)	5,408,044 (38.92)
Alabama	60 (5.22)	441,044 (5.48)	14 (1.5)	70,736 (0.51)

^a^NH: non-Hispanic.

^b^MSM: men who have sex with men.

^c^US Census data include those who selected only Black/African American individuals and do not include those who selected multiple races (US Census Bureau. 2019. https://data.census.gov/cedsci/ accessed 2.18.2021).

## Discussion

### Public Health Need

Formative research synthesized concrete guidance from stakeholders and community members who informed the methods used to recruit and retain participants in this study. We were able to effectively engage BMSM and HLMSM in this project and share best practices related to recruiting this key population. BMSM and HLMSM are disproportionately affected by HIV [28], and to achieve health equity, it is necessary to develop methods to maximize the opportunities for these communities to access resources such as HIVST distribution. iSTAMP aimed to learn how to effectively reach BMSM and HLMSM and to implement a program for the distribution of free HIVSTs.

Barriers to HIV testing have been described for decades throughout the HIV epidemic in the United States. These include a lack of perception of risk, stigma associated with seeking testing, lack of convenient options for HIV testing, and fear of obtaining a positive test result. Stigma-related concerns may be heightened for BMSM and HLMSM who face intersectional stigmas around race and ethnicity, sexual or gender minority status, and HIV. It is crucial to develop a robust slate of option for HIV testing that will allow MSM at elevated risk for HIV to test as frequently as recommended by the CDC. One approach is by supplementing provider-based testing with HIV self-testing.

### Key Innovations

Working closely with the states in which the study was conducted was an important aspect of our study. We conducted this study in states with large populations of BMSM, HLMSM, or both [13]. Involving the state health departments is crucial in planning implementation studies of interventions, because in the United States, with a few exceptions, the primary responsibility for providing prevention services and for following up with those living with HIV falls on state health departments. Thus, state health departments may eventually be implementers of the procedures developed and tested in this study. Including states in the planning phases increases the likelihood that programs can be rapidly and seamlessly implemented by health departments that choose to adopt them.

The cost-effectiveness analyses outlined in this protocol will provide necessary information for providers wishing to implement similar recruitment and testing promotion strategies to reach BMSM and HLMSM, among others. Data on both the cost and impact of such strategies can guide decision-making regarding the efficient allocation of limited resources to increase HIV testing among these populations. Although cost-effectiveness analyses are recognized as an important component of implementation science research, limited data on the cost-effectiveness of varied recruitment, HIV testing, and linkage strategies currently exist. Thus, iSTAMP will make an important contribution to HIV testing and prevention services.

### Limitations

Our study had some limitations, including standard biases for randomized controlled trials. The study may be affected by selection bias in that the men who agreed to participate were not representative of all BMSM and HLMSM in the implementation states. Another potential limitation is the differential loss to follow-up between the intervention and control arms. This was mitigated through comprehensive efforts to retain participants in both arms by using multiple modes of contact (phone call, email, or SMS text message), compensating participants for their time to complete study activities, and by allowing all survey and study activities to be completed remotely. There is risk for misclassification bias because we rely, to a large extent, on participant-reported outcomes for the use of tests and linkage to follow-up services. We have taken steps to reduce this bias by allowing participants to complete surveys remotely, using survey tools that have been validated [9], and by measuring key outcomes from multiple sources of data (ie, surveys and communications with the staff). One factor that might influence the results is that this study was primarily conducted during the COVID-19 pandemic [29,30]. A study conducted in 2020 found that during the COVID-19 pandemic, MSM reported fewer sex partners and opportunities to have sex, as well as problems accessing HIV testing, which might have increased the use of HIVSTs [30]. However, because the study had been designed to be conducted entirely remotely, the procedures and protocols for the iSTAMP study did not meaningfully change due to the COVID-19 pandemic.

### Public Health Implications

The distribution of HIVSTs is already moving into public health programs. It is critical that we identify and disseminate effective methods to reach BMSM and HLMSM through programs that strive toward health equity for these priority populations. A pilot project in which a community organization partnered with state and local health departments to fulfill orders for free HIVSTs showed that providing HIVST in a “direct-to-consumer” model is feasible and can reach populations with substantial HIV risk and those who have not recently tested for HIV [31]. In 2021, the CDC contracted with Insignia Federal Group, who partnered with Building Healthy Online Communities and Emory University to distribute 100,000 HIVSTs to those who requested an HIVST in the United States or Puerto Rico [32]. The marketing strategy was primarily driven by social media advertising and influencers to reach Black and Latino gay and bisexual cisgender men, transgender women, and Black cisgender women [32] and also included links to the HIVST ordering portal through the HIV testing locator of AIDSVu.org [11]. It is important that these programs continue to monitor the distribution of HIVSTs in the populations most affected by HIV. In the longer term, local or state health departments and CBOs that serve as major sources of public health guidance and provision of services could implement HIV self-testing programs that serve their local populations most affected by the HIV epidemic. The lessons learned in the iSTAMP study will be useful for the planning and budgeting of programs, for anticipating the need for telehealth counseling and linkage services, and for providing examples of program materials to help jump start national, state, or local programs.

## Appendix

Multimedia Appendix 1 Peer reviewed report by National Center for HIV, STD, and TB Prevention Special Emphasis Panel (National Institutes of Health, USA).

## Abbreviations

BMSM: Black or African American, non-Hispanic men who have sex with men
CBO: community-based organization
CDC: Centers for Disease Control and Prevention
CRF: case report form
DBS: dried blood spot
HIPAA: Health Insurance Portability and Accountability Act
HIVST: HIV self-test
HLMSM: Hispanic or Latino men who have sex with men
ICER: incremental cost-effectiveness ratio
iSTAMP: Implementation of Rapid HIV Self-Testing Among Men Who Have Sex With Men
LGBTQ: lesbian, gay, bisexual, transgender, and queer
MSM: men who have sex with men
PrEP: pre-exposure prophylaxis
STI: sexually transmitted infection

## Data Availability

After the primary outcome analyses are completed, deidentified data sets can be made available upon written request to the investigator PSS. Interested parties should propose a specific analytic hypothesis. The data use agreement would be developed to ensure (1) data are used solely for research purposes without attempting to identify individual participants; (2) that there is a clear plan for maintaining the safety and security of data; and (3) commitment to the destruction or return of data after analysis.
