# Supplementary material for: Engaging Black or African American and Hispanic or Latino Men Who Have Sex With Men for HIV Testing and Prevention Services Through Technology: Protocol for the iSTAMP Comparative Effectiveness Trial
Source: JMIR Res Protoc. 2023 Jan 6;12:e43414. doi: 10.2196/43414 (PMC9862319; doi:10.2196/43414)
Supplement: Multimedia Appendix 1 [file resprot_v12i1e43414_app1.pdf]

SUMMARY STATEMENT
(Privileged Communication)

PROGRAM CONTACT:
PHD Paul Smutz
770-488-1508
wsmutz@cdc.gov

Release Date: 03/29/2017
Revised Date:

Application Number: 1 U01 PS005181-01

Principal Investigator

SULLIVAN, PATRICK SEAN

Applicant Organization: EMORY UNIVERSITY

Review Group: ZPS1 GCA (27)
National Center for HIV, STD, and TB Prevention Special Emphasis Panel

Meeting Date: 02/22/2017
RFA/PA: PS17-003
Council: JAN 2017
Requested Start: 09/01/2017

Project Title: Engaging African American and Latino MSM for HIV Testing and Prevention Services through Technology
SRG Action: Impact Score:30

Human Subjects: 30-Human subjects involved - Certified, no SRG concerns
Animal Subjects: 10-No live vertebrate animals involved for competing appl.
Gender: 3A-Only men, scientifically acceptable
Minority: 2A-Only minorities, scientifically acceptable
Children: 1A-Both Children and Adults, scientifically acceptable

Project Year	Direct Costs Requested	Estimated Total Cost
1	531,849	831,209
2	548,400	857,076
3	548,622	857,423
4	257,413	402,302
TOTAL	1,886,284	2,948,011

1U01PS005181-01 SULLIVAN, PATRICK

BUDGET NOTE

RESUME AND SUMMARY OF DISCUSSION: This application, by a seasoned Principal Investigator (PI), proposes to develop formative research to inform culturally sound recruitment and HIV testing promotion strategies for Black and Latino men who have sex with men (MSM); assess the effects of two innovative strategies that combine online recruitment and testing promotion strategies to increase HIV testing among Black and Latino MSM; and outline ways in which health departments and community-based organizations (CBOs) may adopt these strategies to improve their services to these communities. Reviewers determined this application to have high potential for public health impact. The significance of the proposed work is demonstrated in that Black and Latino MSM have been affected disproportionately by HIV / AIDS since the beginning of the epidemic. Increased testing is needed to reduce HIV rates significantly in these communities. If successful, the results of the project are highly likely to contribute much needed knowledge to assist health service providers and clinicians in delivering innovative and culturally-relevant HIV testing and follow-up treatment services to Black and Latino MSM who test positive. Major strengths of the application identified by reviewers include: An outstanding PI with greater than 20 years of experience in HIV prevention and epidemiology work with MSM, including web- and app-based recruitment for MSM in general; a competent and seasoned research team of co-investigators and other colleagues with complementary and integrated expertise in relevant areas; the team's demonstrated experience and expertise to design, implement, and evaluate the proposed study; provision of extremely well-detailed and impressive biosketches; the team's experience in obtaining the required data security systems and certifications meeting Federal Information Security Modernization Act (FISMA) requirements; the very well-written and organized nature of the application; well-conceived overall strategy, methodology, and analyses that are based appropriately upon the team's prior research; provision of compelling evidence to substantiate the public health significance of the proposed effectiveness study; stated use of the Social Cognitive Theory and the Integrated Model of Behavior Theory as theoretical bases for the study; well-detailed plans for recruiting Black and Latino MSM; availability of web-based recruitment strategies in both English and Spanish; clearly described plans for pilot testing the recruitment, enrollment, and data collection components of the study; good strategies to prevent a respondent from enrolling more than once in the study; adequately described retention strategies that are based upon previous virtual and local cohort studies; integration of a telemedicine component, which has not been used in a comparative effectiveness study; well-planned, detailed, and adequate power analyses; provision of a well-developed logic model that includes inputs, outputs, and outcomes of the study organized by study year; clearly described plans for participants with positive test results to receive help with linkage to care by setting up appointments at the provider of their choosing; nicely detailed discussion of potential problems, alternative strategies, and benchmarks of success; and outstanding scientific environment with abundant resources and institutional support.

Weaknesses identified by reviewers include: The team's minimal experience working specifically with Latino MSM populations; the number of investigators and consultants devoting very small percentages of their time; failure to cite any of the prior literature focused specifically on Black or Latino MSMs; some concern that no theoretical concepts will be advanced in that although it is stated that the proposed study is framed in Social Cognitive Theory, this theory is not integrated into the methodology and analysis; multiple study components (apps, telemedicine, qualitative research, self-testing at home, and dried bloodspots) which are complex and have potential for multiple simultaneous errors; uncertainty regarding whether simply translating the two established apps into Spanish will be sufficient to be culturally effective; concern that many Spanish-speaking MSM may not have sufficient literacy skills to interact with the apps, which could eliminate a key population that is often absent from HIV prevention research; inadequate description of process evaluation data collection and measures to

ensure study integrity; insufficient budget considerations in the evaluation plan; very few details about where Black and Latino MSM will be referred to for local HIV-related services and an insufficient description of the types of services such as additional HIV testing, partner services, pre-exposure prophylaxis (PrEP), and other related services; no evidence of prior arrangements with known HIV care providers / clinics, and reporting of linkage to care based on self-report; and the stipulation that participants must have access to a private computer with internet access, which may be difficult in lower socioeconomic status (SES) minority men. Protection of human subjects is adequately addressed. Inclusion is acceptable in terms of gender, minority groups, and children. There are no animal welfare or biohazard issues. There is some concern with respect to the budget in that the timeframe for the formative and implementation periods, and steps in the logic model, do not align with the budget over the proposed four years of the project. Overall, this is an excellent application that is very strong with only some minor weaknesses.

DESCRIPTION (provided by applicant): Men who have sex with men (MSM) are a key risk group in the United States and are disproportionately impacted in terms of HIV prevalence. The disparities experienced by MSM, and especially by Black and Hispanic MSM, are explicitly recognized in the National HIV AIDS Strategy, which calls for reducing disparities experienced by MSM, especially MSM of color. HIV testing is a key component of combination HIV prevention packages. Testing allows men to be aware of their HIV infection status and serves as a stepping stone to additional HIV prevention services, such as pre-exposure prophylaxis. Increasing testing rates among Black and Hispanic MSM is a key component to reducing disparities among these groups. We propose a comparative effectiveness study to assess the effects of two innovative strategies that combine online recruitment and testing promotion strategies to increase HIV testing in Black and Hispanic/Latino MSM. The two innovative strategies will be compared to a traditional recruiting strategy and comparison condition involving providing information about HIV testing and testing locations. The recruitment methods will be a social networking and dating app targeted to MSM (Grindr) and advertisements on gay-related websites; these recruiting strategies will be compared to a non-innovative strategy of targeted Facebook advertising. Innovative testing promotion strategies will be an app-based HIV prevention portal (HealthMindr) and a mobile-optimized website (healthMpowerment). Our primary aims are to conduct formative research to develop culturally appropriate innovative recruiting and testing promotion strategies, conduct a comparative effectiveness study to assess the effectiveness of each strategy in black and Hispanic MSM, and to develop a translation and dissemination plan to support uptake of the resulting intervention strategies in state and local health departments and community-based organizations.

PUBLIC HEALTH RELEVANCE: Black and Hispanic MSM are the most heavily impacted risk groups in the US HIV epidemic, but past studies have illustrated challenges with recruiting these men to uptake routine HIV testing. We propose a comparative effectiveness study to evaluate the relative performance of two strategies – each based on an innovative, established internet-based platform – to promote HIV testing among Black and Hispanic MSM.

CRITIQUE 1

Significance: 1
Investigator(s): 2
Innovation: 1
Approach: 2
Environment: 2

Overall Impact: This is a new application with a seasoned and outstanding Principal Investigator who has over 20 years of experience in HIV prevention and epidemiology work with MSM, along with a

competent and seasoned research team of co-investigators and other colleagues. The application proposes a project with the major goals of developing formative research for culturally-sound recruitment and HIV testing promotion strategies for Black and Latino MSM (BLMSM), assessing the effects of two innovative strategies that combine online recruitment and testing promotion strategies to increase HIV testing in Black and Latino MSM, using Grindr (mobile app) and ads on gay websites versus Facebook ads (for recruitment) plus HealthMindr (an HIV prevention app) and healthMpowerment (a mobile-optimized site) for promotion of HIV testing. The investigators will also be outlining ways in which health departments and CBOs may adopt these strategies to improve their services to these communities. This is a highly significant area because BLMSM have been disproportionately affected by HIV/AIDS since the beginning of the epidemic and testing efforts must increase to significantly reduce HIV rates in these communities. It is a highly innovative application because it will integrate the use of modern technologies and methods for reaching BLSMSM in the most effective ways, including mobile apps to increase engagement in HIV testing and telemedicine counseling, along with customized referrals for men who have seropositive results. The primary outcome is to have participants test for HIV within one month of enrollment, with calculations conducted separately for Black MSM and Latino MSM.

Strengths

- This study application is very well written and organized. Innovation of using multiple recruitment strategies with BLMSM and evaluating the feasibility of each one is key to the success of this project.
- Dr. Sullivan and his team have the experience and expertise to fully design, implement, and evaluate this study.
- There was a lot of evidence provided to ensure a successful completion of the proposed study.

Weaknesses

- The application had a few minor areas that needed improvement, including inadequate specific expertise in research with Latino MSM, very little description of measures to ensure study integrity, and very few details about where BLMSM will be referred to for local HIV-related services.

Significance: The project addresses the fact that Black and Latino MSM (BLSMSM) communities in the US have been disproportionately represented in the modern HIV epidemic with increasing rates of new HIV diagnoses and less likelihood of awareness of HIV infection compared to their non-BLMSM counterparts. This is occurring despite the evidence showing that BLSMSM and White MSM have similar rates of recent testing for HIV.

The project, if successfully completed, will contribute much needed knowledge to assist health service providers and clinicians to provide innovative and culturally-relevant services to BLMSM, related to HIV testing and follow-up treatment (for those who test positive).

Successful completion of the aims will provide evidence needed to significantly improve recruitment strategies to find BLMSM in highly affected areas of the United States, engage them in HIV testing efforts, and link participants with HIV to the medical and support services they need. The innovations in recruitment and the technology proposed by the investigators, by using mobile apps/sites, like Grindr, Facebook, and gay-related sites, will reach more BLMSM and, because of the comparative nature of the study, will provide much needed information on the most effective and cost-efficient ways to reach BLMSM in culturally-respective ways.

The investigators report recent unpublished data they developed showing how risk of new HIV infections is mainly concentrated in the US South and coastal areas. In addition, they stated and provided evidence to show that the areas of highest impact are different for Black and Latino MSM. Thus, the investigators will focus their study on improving HIV testing among Black MSM in NC, SC, GA, AL, MS, FL, and LA, as well as among Latino MSM in CA, NV, MS, LA, TX, NY, and FL.

The application contained well-written, specific, and concise reporting of HIV testing and prevention services for BLMSM as well as HIV treatment services (*see Table 1 and its corresponding narrative*). The investigators stress that efforts in reaching BLMSM cannot focus solely on engaging them to adopt PrEP; recruitment into testing services will link them to needed services including PrEP as well as medical services for HIV positives and referrals to partner services. The investigators presented this information within the text of the application as well as in tabular form. As shown in Table 1, on page 166, they showed how there are “opportunities for improvement” for linking BLMSM to testing and linkage to care services.

Strengths

- The investigators provided more than enough evidence to substantiate the public health significance of conducting this effectiveness study.

Weaknesses

- There are no weaknesses to report for this section.

Investigator(s): Over 50 years of collective experience is represented on this research team. Dr. Sullivan has assembled an exemplary group of colleagues and other collaborators to carry out this important project. He is joined by colleagues at Emory who have experience in HIV epidemiology as well as RCTs of HIV prevention interventions (e.g., Drs. Rosenberg and Wall). In addition, experts in Black or Latino MSM HIV prevention (e.g., Greg Millett, Latesha Elorpe, Kenneth Mayer) and collaborators at the University of Michigan and the University of North Carolina have been included to further add credibility and necessary expertise for implementing this project.

The members of the research team have each documented their achievements and accomplishments in their biosketches which are well detailed and exemplify expertise in the field.

Dr. Sullivan is the PI of this proposed project. There are six (6) co-investigators and multiple consultants. The research team members definitely show that they have complementary and integrated expertise in HIV prevention, treatment, medical and support services, as well as epidemiological research. This is described in detail in section 4.2 (and Figure 3) of the application.

All project personnel are well qualified to conduct work on this project. Considering the multi-site nature of the recruitment and testing aspects of this project, the percentages of time presented in the application seem adequate. In addition, the PI, the Co-I’s, and the consultants/collaborators all have experience in multicultural and/or structural approaches to HIV prevention and/or treatment. They also show, according to their biosketches, successful acquisition of grant funding and completion of research studies with high public health impact and relevance to the work proposed in this application.

Dr. Sullivan has very recent experience in research with young Black MSM (1R01DA038196) and with racial disparities in HIV care (1R01AI112723). His colleagues have excellent track records in conducting formative research in HIV prevention with YMSM, Black MSM, and other MSM populations and disenfranchised communities.

Dr. Sullivan has an excellent track record in web-based and app-based recruitment for MSM in general. His specific expertise is not in conducting research with adult BLMSM populations; however, he has complemented his expertise with that of others who have worked with the BLMSM populations (i.e., Greg Millett, Latesha Elorpse, among others).

The research team has shown that, collectively, it has the expertise to conduct online HIV-related research with MSM in general, specifically YMSM and Black MSM. Work specifically with Latino MSM is shown only through collaboration with Dr. Hightow-Weidman; otherwise, no other specific expertise in Latino MSM research is shown in the application, despite the details provided in Table 2. Most of the reported work with Latino MSM seems to be conducted as part of other research among MSM.

Based on the multiple publications and grants reported and referred to in the application, Dr. Sullivan and his assembled team of researchers and collaborators have the ability to collect, manage, and analyze data in a timely manner.

The research team also seems to have recent experience in obtaining the required data security systems and certifications meeting federal FISMA requirements.

Strengths

- The research team has a substantial amount of experience with formative research and recruitment work with MSM populations, especially Black MSM.

Weaknesses

- However, the team only has minimal experience working specifically with Latino MSM populations.

Innovation: Dr. Sullivan and his research team propose a study that will challenge current web-based recruitment and linkage to services paradigms in five ways, as stated in the application. Among those approaches stated are 1) comparing two recruitment strategies for BLMSM against a more common (comparison) strategy (i.e., Facebook ads) as well as 2) offering telemedicine for counseling participants in both intervention arms within a comparative effectiveness study.

Though some aspects of the study have been used before by the researchers and in HIV prevention research, the combination of the approaches (i.e., using web-based apps) and the context in which the research is being conducted (i.e., multi-site, with BLMSM) is novel in the field of HIV prevention and linkage to care research.

The investigators are proposing that they will be using Social Cognitive Theory and the IOM Integrated Model as theoretical bases for the study; thus, results of the study will refine and improve the applicability of these theories, especially as they apply to the online behavior of BLMSM communities related to HIV testing and prevention.

The application describes a detailed plan for recruiting BLMSM using Grindr and gay websites (vs. Facebook ads). Each of the 8 consultants on the project will be tapped to provide expertise on how to develop these recruitment strategies in the most effective ways.

Dr. Sullivan is basing the proposed work on prior research conducted by him and his research colleagues; however, this is not duplication of current or prior research.

As documented in section 4.3.6, the investigators plan to provide information and referrals to services in a cost-effective manner.

The research team states in section 4.3.1 that "there is sufficient evidence from [their] work and others' that simply recruiting men and either recommending that they test for HIV or offering HIV test kits is not sufficient to produce optimal uptake of testing, especially for MSM of color." With this in mind, they provide a rationale for identifying and developing innovative strategies to increase testing among BLMSM. In addition, as they mentioned in Section 3, the need for internet based approaches is needed for "reaching large numbers of men at limited marginal costs" (p. 167).

Strengths

- The proposed study has two innovative recruitment strategies being tested against "standard" Facebook ad recruitment.
- Online recruitment and HIV testing promotion (via telemedicine) in several areas of the US strengthens the innovation aspect of the study as well the public health significance.

Weaknesses

- There are no weaknesses to report for this section.

Approach: The strategies explained in the application are sound and well thought out. The methodology and analyses also seem appropriate for this project. The use of 8 consultants at multiple sites across the country may seem less community-driven than a project of this kind should integrate; however, because cost-effectiveness is a key component of this project, the use of multiple consultants is appropriate. Also, they plan to obtain a sample of 60 BLMSM to do online focus groups to gather feedback on the HIV testing promotion strategies of the study. Because the app and web-based strategies have already been normed successfully with MSM, the proposed project seems to utilize approaches that will be potentially successful in recruiting BLMSM from multiple sites across the US.

The researchers have presented a detailed discussion of possible problems and alternatives as well as benchmarks to measure success in the project.

The investigators have outlined a well-detailed plan on how to conduct this effectiveness study as well as how to manage the riskiest aspects of the study. They mentioned in section 4.4 how they successfully managed the development of HealthMindr and healthMpowerment over the past several years, including issues related to advancements in technology.

The study team plans to recruit and enroll a total sample of up to 3600 BLMSM. They provided details on human subjects protection (HSP) for every aspect of the study, including online focus group discussions with 60 BLMSM as well as the participants in the RCT. They also discussed HSP for participants ages 18-21, including special considerations made for this age group.

Section 4.5 presents a well-written logic model including several inputs, outputs, and outcomes of the study organized by study year.

The investigators describe their plans for recruiting 1800 Black MSM and 1800 Latino MSM for the study, which meets the required total, as described on page 6 of the FOA.

They also describe in much detail how they plan to address all four goals of the FOA. It was very helpful to review plans to develop and evaluate their web-based recruitment strategies for BLMSM in

both English and Spanish, utilizing Spanish-speaking staff on the team (through its collaboration with the University of Michigan) for culturally-relevant Spanish translations of their apps and recruitment materials.

The PI and his team describe how they will engage the target population in these recruitment strategies with their online focus groups discussions (FGDs), with 60 men via 10 FGDs.

The investigators, in section 4.3.3., provide details for implementing the strategies and to pilot test the recruitment, enrollment, and data collection aspects of the study.

They also fully describe how they will consent, screen, and enroll participants using SurveyGizmo and keep the data on a HIPAA-compliant server at Emory University.

The research plan, study design, and power analyses are well-planned, detailed, and adequate. Their power analysis calculations assume a 40% uptake of testing within one month of study enrollment in the comparison arm while detecting 10% increase in testing in the intervention arms for each group (Black MSM and Latino MSM).

They also mention, in section 4.3.5, how they plan to examine test result outcomes, linkage to care, and linkage to additional services, and implement referral to HIV testing, PrEP, nPEP, health insurance, mental health services, and other related services.

The investigators also describe how they plan to recruit and follow-up with participants, based on the PI's and colleagues' past experiences in conducting research studies with similar populations.

As stated in section 4.3.5, the investigators will conduct a pilot study to test recruitment, data collection, and enrollment systems. This includes the Spanish-translated versions of materials for Grindr, HealthMindr, healthMpowerment, and the Facebook ads (comparison intervention).

The application mentions that Dr. Wall (one of the Co-I's) will be "responsible for measuring cost of recruitment strategies and cost-effectiveness analyses" (p. 168 and p. 180); in addition, in section 4.3.6, Dr. Sullivan and his team state that they will utilize "standard methods of cost analyses, as recommended by the US Panel on Cost-effectiveness in Health and Medicine."

The investigators state that participants with positive test results will receive help for linkage to care with setting up appointments "at the provider of their choosing, using a list of providers in the study states who provide HIV care from [their] AIDSVu.org national resources and the American Academy of HIV Medicine provider locator" (section 4.3.3.13).

Regardless of the arm of the study that participants are in, each participant will be administered a baseline survey requesting information on "HIV testing history, HIV testing knowledge and beliefs, current HIV testing strategies, last HIV test and results, intentions/plans to test for HIV in the future, use of other HIV prevention services, and sexual risk and preventive behaviors" (p. 176).

The investigators mention that they will collect follow-up data from participants in the RCT approximately four months after enrollment with a $25 incentive to assess completion of HIV testing within one month of enrollment. Anyone who returns proof of HIV testing will receive an additional $10 incentive via Amazon gift credits (p. 177).

The investigators also describe how HIV testing will be tracked by recording the participant's reported HIV test result as well as tracking referrals and linkages (p. 177-179).

All data collection activities, including assessment of recruitment costs, were described. The investigators were very succinct yet detailed in discussing a study design with enough flexibility for making a few modifications, if necessary, to successfully and ethically complete the study (see section 4.4). In addition, a detailed logic model and realistic timeline was provided. The proposed activity was scalable (see section 4.5). Even though a well-developed logic model was provided, no specific evaluation questions or items were provided related to budget, except for brief mentions of "budget" in Table 4.

There did not seem to be specific information relating to collecting process evaluation data to assess contextual factors that may relate to changes in outcomes, as described in the FOA, nor the use of Evaluation Standards for judging the quality of the study.

The research plan in this application provides details regarding the collection of outcome data as described in the FOA (e.g., number of Black and Hispanic/Latino MSM tested for HIV within one month of enrollment and provide the result of the test). It is summarized very well in Table 4 (p. 181).

The research team expects 128,925 BMSM and 522,531 HLMSM to be active on any one of the recruitment platforms in the states they are recruiting from; thus, the efforts described in the application have potential to reach a large proportion of the population.

The application includes an adequate plan for verifying results of HIV RDT and locally conducted HIV testing, as described in section 4.3.3. In addition, there is a description of how results will be provided to participants for tests conducted at the CDC and local labs.

The investigators describe in detail why the proposed study design, including sample size, power, and effect size, will guide them to successful completion of the study. The quality of the proposed research plan, study design, and power analyses adequately address the research objectives of the FOA (see section 4.3). As mentioned before, the investigators propose an adequate sample size calculation (of 600 men in each arm of the study).

The investigators briefly but adequately describe retention strategies based on previous virtual and local cohort studies to achieve retention rates of at least 70% (see section 4.3.3.6).

Quality assurance will be led by Dr. Jones (Co-I) (see section 4.3.4.6). In addition, Ms. Luisi, a data manager for the project will assist with QA (p. 125). However, the investigators did not explain in detail measures that ensure integrity of online systems, enrollment of study participants, delivery of home testing materials, and retention of study participants.

Strengths

- The approaches described were well written, with adequate details that provided evidence for the potential of a successful research project.

Weaknesses

- Description of measures to ensure integrity of certain aspects of the study (i.e., enrollment, home testing delivery) and process evaluation data collection were not provided in an adequate manner.

- Only passing mentions about budget considerations were included in the evaluation plan.

Environment: Dr. Sullivan and his research team have the scientific expertise and are in environments conducive to the completion of successful research studies. Among all the institutions represented by the study team members (e.g., Emory, UNC, UM, among others mentioned), there is more than adequate support and related resources reported to implement and complete the study.

The collaborations being established in the study will help ensure the completion of the study, especially because of its multi-site arrangement throughout several states.

The investigators report that they will have access to adequate numbers of Black and Latino MSM by virtue of the collaborators and consultants located in the several states they will be recruiting from. In addition, Spanish-speaking staff will help reach Spanish-speaking Latinos for potential recruitment into the study.

The application, in the budget justification section and other areas, provides detailed descriptions of duties and responsibilities of project personnel; a summary of personnel duties is summarized in Figure 3 (p. 168).

The plans put forth in the application for programming online systems, data management, data security, and data analyses is adequately described and the data security systems are sufficient to protect participants' data and confidentiality, as evidenced by the research team's past and current study implementation experiences (see section 4.3.4).

The application includes a detailed total budget with staffing plans and activities for each project year.

The research team, however, did not perform an adequate job in describing the local services for referring study participants for additional HIV testing, partner services, PrEP, and other related services.

Strengths

- More than 50 years of collective experience exist among the main members of the research team.
- The collaborations and institutional/community environments will provide a forum for a potentially successful study.

Weaknesses

- There were very few details about the agencies BLMSM will be referred to for local HIV-related services.

Protections for Human Subjects:

Acceptable Risks and/or Precautions

Vertebrate Animals:

Not Applicable (No Animals)

Biohazards & Select Agents:
Not Applicable

Budget and Period of Support:
Recommend as Requested

CRITIQUE 2

Significance: 1
Investigator(s): 2
Innovation: 1
Approach: 4
Environment: 3

Overall Impact: This is a new application with an outstanding Principal Investigator with the major goal to evaluate the performance of two internet-based strategies to promote HIV testing among Black and Hispanic MSM. The first step will be a formative phase to assess the two internet-based strategies and refining two existing web-based tools through theater testing and qualitative interviews. The two strategies will include a testing promotion component, HealthMindr, a mobile phone app and healthMpowerment, an HIV education interactive web app. Next, they will conduct a comparative effectiveness study of the two internet-based strategies in comparison to recruitment through Facebook. The primary outcome is testing for HIV within the first month of enrollment. Finally, they will conduct a secondary outcomes analysis. This is a highly significant area because young Black and Latino MSM are disproportionately affected by HIV and innovative ways to increase testing, condom uptake and prevention strategies to engage these key populations are needed. This is a highly innovative application because they will use a hybrid comparative effectiveness design which allows for the possibility that different post-recruitment modalities of testing promotion are better for men recruited through different internet-based formats; they will test two app based strategies HealthMindr based on Social Cognitive Theory and healthMpowerment based on the IOM's Integrated Model of Behavior Theory; and the two prevention platforms will potentially be able to reach a large audience for a relatively small cost.

Strengths

- Use of Social Cognitive Theory which will focus on the importance of self-regulation in learning via the HealthMindr.
- Use of Integrated Model of Behavior Theory, which has been widely used as a tool to design and evaluate health behavior change interventions via healthMpowerment.
- This is an extremely seasoned study team with a successful independent and collaborative experience well suited to complete this project.

Weaknesses

- Requires participants to have access to a private computer with Internet access. This may be difficult in lower SES minority men.
- Not sure if simply translating the two established apps into Spanish will be enough to be culturally effective.

Significance:

Strengths

- By focusing on Black and Latino MSM this study is focusing on the racial and ethnic groups that are most disproportionately affected by HIV.
- The primary outcome is HIV testing within 1 month of enrollment, which along with increased frequency of testing will allow for a greater number of Black and Latino MSM to be aware of their HIV status.
- The study aims to help Black and Latino MSM recognize and assess their personal risks and to provide them skills to develop and adhere to risk-appropriate plans for regular HIV testing. Understanding one's risk factors is key to increasing HIV testing.
- The study focuses on improving HIV testing in the geographical areas that have the highest rate of new HIV infections in Black and Latino MSM.
- By evaluating the effectiveness of app based strategies to increase testing, this will provide a tool to reach MSM at the highest risk for HIV acquisition at relatively low cost.

Weaknesses

- None noted.

Investigator(s):
Strengths

- The study PI and site PI's have successful, recently competed, and ongoing research involved with different but complementary strengths in the areas of technology, telemedicine, implementation of online interventions, HIV prevention, epidemiology, biostatistics, mail-out HIV testing kits for MSM, cost analysis and qualitative research.
- The investigators have included multiple members of the study team that have worked with MSM as well as have at least one member that is a native Spanish speaker.

Weaknesses

- None noted.

Innovation:
Strengths

- The investigators will integrate a telemedicine component, which to date has not been used in a comparative effectiveness study, in both strategies to ensure proper self-administration of OraQuick or self-collection of DBS specimens
- By using existing knowledge of internet based recruitment, it focuses on HIV testing uptake and linkage to HIV care or prevention services by using and improving on existing online modalities.
- The study is comparing two strategies on two different theoretical bases: Social Cognitive Theory and Integrated Model of Behavior theory.

Weaknesses

- None noted.

Approach:
Strengths

- The investigators will capitalize on preliminary data on HealthMindr and healthMpowerment developed by the PI and Co-PI (Sullivan and Stephenson).
- The investigators will translate both apps into Spanish.

- As smart phones are ubiquitous in the US, with use greatest in youth and young adults, this study has the potential to reach its targeted population at a relatively low cost.
- The protocol of self-administered OraQuick and DBS is well described and will allow for a greater accuracy than simple self-reporting of HIV testing.

Weaknesses

- The study consists of multiple components: apps, telemedicine, qualitative research, self-testing at home and DBS, which are complex and have the potential for multiple simultaneous errors.
- Not sure that translation from English to Spanish will be culturally appropriate for Spanish speaking MSM. Although the investigators include Spanish speakers with experience in HIV prevention, not sure this will be enough to reach this audience.
- Many Spanish speaking MSM may not have sufficient literacy skills to interact with the apps. Thus, eliminating a key population that is often lost or omitted in HIV prevention research.
- The study requires that participants have private access to a computer with internet access which may be difficult in both the targeted populations.
- Secondary outcomes rely heavily on surveys/self-report, introducing missing and false reporting. However, this was a requirement of the FOA.

Environment:
Strengths

- The primary location (Emory) and sites (Univ. of Michigan, Univ. of North Carolina Chapel Hill) as well as recruitment clinics (UNC, CAPUS Men's clinic, Wake County Human Services, Durham County Dept. of Public Health STD clinic) are all well equipped with institutional support and physical resources to ensure success.
- The seven states are ideal for their targeted population (BMSM: NC, SC, GA, AL, MS, FL and LA and for HMSM: CA, NV, MS, LA, TX, NY and FL)

Weaknesses

- Referral to prevention and HIV care services is described, mostly consisting of online resources, and there is no prior arrangements with known HIV care providers/clinics. Reporting of linkage to care will be completely based on self-report.

Protections for Human Subjects:

Acceptable Risks and/or Precautions

Vertebrate Animals:

Not Applicable (No Animals)

Biohazards & Select Agents:

Not Applicable

Budget and Period of Support:

Recommend as Requested

CRITIQUE 3

Significance: 4
Investigator(s): 4
Innovation: 4
Approach: 4
Environment: 4

Overall Impact: This is a new application from a very good Principal Investigator with three goals. The first is to conduct a formative phase to assess two innovative, culturally-appropriate and sensitive, internet based strategies to recruit BMSM and HLMSM to HIV testing. This will include translation into Spanish, expert review, and engagement of men in research communities to refine existing web-based tools (healthMpowerment and HealthMindr) through theater testing and qualitative interviews. The second goal is to conduct a comparative effectiveness study of two innovative strategies with a third, non-innovative comparison strategy to promote uptake of HIV testing by Black and Hispanic/Latino MSM. 3,600 MSM will be enrolled attaining at least 70% retention over 4 months, with a primary outcome of HIV testing within 1 month of enrollment. The third goal is to conduct secondary analyses of outcomes of relevance to the HIV prevention and care cascades, including linkage to HIV care or prevention services and costs per person tested for HIV, diagnosed with HIV, linked to medical care, linked to prevention or social services. This is a significant area because HIV risks and infections are continuing vectors of the AIDS epidemic with potential to renew the outbreak of HIV in the general public. It is a highly innovative application because HIV testing has not been done before in this way with these high-risk populations.

Strengths
- The investigative team is very good.
- This is a clearly innovative project.
- The facilities and resources for doing this project are very good.

Weaknesses
- The team does not describe what a culturally-appropriate approach is for African-American and Latino MSMs.
- This work is not theory-based, despite mention of social cognitive theory and may not advance the field or science.
- It is not clear that the PIs will devote enough time to this project. Managing the large number of co-investigators and consultants will be a project unto itself, apart from the research.

Significance:
Strengths
- This is a new and potentially cost efficient approach to HIV testing, access to treatment and prevention.
- Recruitment via the internet and social media is an expansion of technical capacity.
- The application justifies the proposed recruitment jurisdictions and provides evidence on HIV testing prevalence, engagement in prevention services, partner services, PrEP, and HIV treatment among black and Hispanic/Latino MSM.

Weaknesses

- The approach via internet and social media may not improve scientific knowledge or clinical practice.
- It is not clear that use of this medium (internet) will change concepts, treatment and services.

Investigator(s):
Strengths

- The PD/PIs have an ongoing record of accomplishments, have complementary and integrated expertise, and have demonstrated that they can manage the organizational structure appropriate for such a project.
- The investigator team has the ability to collect, manage and analyze data in a timely manner. They have recent experience in obtaining the required data security systems and certifications meeting federal FISMA requirements.

Weaknesses

- It is not clear that the PI and Co-investigators will devote a sufficient percentage of their time to this project.
- The number of investigators and consultants devoting very small percentages of their time is a concern that the overall project will not receive the attention and quality of effort that it needs.
- The investigators do not define and elaborate on the cultural approaches necessary to do successful HIV prevention and treatment of African-American and Latino MSMs.

Innovation:
Strengths

- The application describes a recruitment strategy plan that includes MSM dating sites or apps.
- The investigators' plan is innovative. No other internet strategy like the one they propose exists for providing Black and Hispanic/Latino MSM HIV testing.
- The application documents that this is not a duplication of current or prior research.
- The application describes a cost-effective mechanism for providing information, referrals and linkage services to study participants.
- The application includes a rationale for identifying innovative, internet-based recruitment strategies for engaging Black and Hispanic/Latino MSM in HIV testing.

Weaknesses

- This is not a theory-based application. Social cognitive theory is offered, but it is not integrated into the methodology and analysis. Therefore, theoretical concepts may not be advanced.

Approach:
Strengths

- The application includes a logic model that specifies research components and the expected outcomes. It also describes how many Black MSM and Hispanic/Latino MSM will be recruited and the baseline assessments to be completed during the recruitment period proposed.
- This application meets all of the requirements of the FOA.

- Provisions have been made to prevent a respondent from enrolling more than once in the study.

Weaknesses

- There is no way to determine whom among Black and Latino MSM will be recruited via internet social network sites or what proportion of Black and Latino MSMs will be reached. None of the prior literature cited focused specifically on Black or Latino MSMs.

Environment:

Strengths

- The people and facilities are more than adequate to do this project.

Weaknesses

- Duties and responsibilities are all spelled out as well as plans for data management, security and data analyses. The number of people (17) and their roles raises the potential that no one will spend enough time on the project to produce a quality product or have a sense of the project's overall status.
- The timeframe for the formative and implementation periods, and steps in the logic model, do not fit the budget over the proposed four years of the project. These disparities are not acknowledged or explained in the budget justification.

Protections for Human Subjects:

Acceptable Risks and/or Precautions

Vertebrate Animals:

Not Applicable (No Animals)

Biohazards & Select Agents:

Not Applicable

Budget and Period of Support:

Recommend as Requested

THE FOLLOWING SECTIONS WERE PREPARED BY THE SCIENTIFIC REVIEW OFFICER TO SUMMARIZE THE OUTCOME OF DISCUSSIONS OF THE REVIEW COMMITTEE, OR REVIEWERS' WRITTEN CRITIQUES, ON THE FOLLOWING ISSUES:

PROTECTION OF HUMAN SUBJECTS: ACCEPTABLE

INCLUSION OF WOMEN PLAN: ACCEPTABLE

INCLUSION OF MINORITIES PLAN: ACCEPTABLE

INCLUSION OF CHILDREN PLAN: ACCEPTABLE

COMMITTEE BUDGET RECOMMENDATIONS: The budget was recommended as requested; however, there is some concern with respect to the budget in that the timeframe for the formative and implementation periods, and steps in the logic model, do not align with the budget over the proposed four years of the project.

MEETING ROSTER
National Center for HIV, STD, and TB Prevention Special Emphasis Panel

NATIONAL CENTER FOR HIV, VIRAL HEPATITIS, STDS AND TB PREVENTION

ZPS1 GCA (27)
02/22/2017

CHAIRPERSON(S)

FERNANDEZ, MARIA ISABEL, BA, MA, PHD
PROFESSOR
NOVA SOUTHEASTERN UNIVERSITY
2000 S. DIXIE HIGHWAY
SUITE 108
FT LAUDERDALE, FL 33314

MEMBERS

BOWSER, BENAJMIN PAUL, BA, PHD
PROFESSOR
RETIRED
7075 ELVERTON DRIVE
OAKLAND, GA 94611

CADOFF, EVAN, BS, MS, MD
PROFESSOR
MONTEFIORE MEDICAL CENTER
111 EAST 210TH STREET
C303
BRONXVILLE , NY 10467

GALLETLY, CAROL L ESQ, PHD, JD
ASSOCIATE PROFESSOR
MEDICAL COLLEGE OF WISCONSIN
2071 NORTH SUMMIT AVENUE
MILWAUKEE, WI 53202

KEESHIN, SUSANA WILLIAMS
ASSISTANT PROFESSOR
UNIVERSITY OF UTAH
295 CHIPETA WAY, 2S010
SALT LAKE, UT 84132

KUO, CAROLINE CHIA, BA, MPHIL, PHD
ASSISTANT PROFESSOR
BROWN UNIVERSITY
121 SOUTH MAIN STREET
BOX GS-121-4TH FLOOR
PROVIDENCE, RI 02903

NANIN, JOSE EDUARDO, EDD, MA, MS
ASSISTANT PROFESSOR OF COMMUNITY HEALTH
DIRECTOR OF EDUCATION AND TRAINING
CENTER FOR HIV EDUCATION AND TRAINING
KINGSBOROUGH COMMUNITY COLLEGE AND HUNTER COLLEGE
2001 ORIENTAL BLVD
BROOKLYN, NY 11235

REBCHOOK, GREGORY MICHAEL, PHD
ASSISTANT PROFESSOR
UNIVERSITY OF CALIFORNIA, SAN FRANCISCO
550 16TH STREET
3RD FLOOR
SAN FRANCISCO, CA 94143

SCIENTIFIC REVIEW OFFICER

ANDERSON, GREGORY
CENTERS FOR DISEASE CONTROL AND PREVENTION
NCHHSTP
1600 CLIFTON ROAD NE, MS E-60
ATLANTA, GA 30329

MORRISON, CHRISTINE J
SCIENTIFIC REVIEW ADMINISTRATOR
OFFICE OF EXTRAMURAL RESEARCH
OFFICE OF THE ASSOCIATE DIRECTOR FOR SCIENCE
CENTERS FOR DISEASE CONTROL AND PREVENTION
1600 CLIFTON ROAD, MS-E60
ATLANTA, GA 30329

EXTRAMURAL SUPPORT ASSISTANT

WILSON, SHARNELL
GRANTS TECHINCAL ASSISTANT
CENTERS FOR DISEASE CONTROL AND PREVENTION
NCHHSTP
1600 CLIFTON RD., MS-E60
ATLANTA, GA 30329

Consultants are required to absent themselves from the room during the review of any application if their presence would constitute or appear to constitute a conflict of interest.
